# Electro-Kinetic Instability in a Laminar Boundary Layer Next to an Ion Exchange Membrane

**DOI:** 10.3390/ijms20102393

**Published:** 2019-05-14

**Authors:** Pierre Magnico

**Affiliations:** Aix Marseille Univ, CNRS, Centrale Marseille, M2P2 UMR 7340, 13451 Marseille, France; pierre.magnico@univ-amu.fr; Tel.: +33-04-13-55-40-69

**Keywords:** ion exchange membranes, electro-convective instability, overlimiting current, concentration polarisation, particle tracking

## Abstract

The electro-kinetic instability in a pressure driven shear flow near an ion exchange membrane is considered. The electrochemical system, through which an electrical potential drop is applied, consists in a polarization layer in contact with the membrane and a bulk. The numerical investigation contained two aspects: analysis of the instability modes and description of the Lagrangian transport of fluid and ions. Regarding the first aspect, the modes were analyzed as a function of the potential drop. The analysis revealed how the spatial distribution of forces controls the dynamics of vortex association and dissociation. In particular, the birth of a counter-clockwise vortex between two clockwise vortices, and the initiation of clusters constituting one or two envelopes wrapping a vortex group, were examined. In regards to the second aspect, the trajectories were computed with the fourth order Runge Kutta scheme for the time integration and with the biquadratric upstream scheme for the spatial and time interpolation of the fluid velocity and the ion flux. The results for the periodic mode showed two kinds of trajectories: the trochoidal motion and the longitudinal one coupled with a periodic transverse motion. For the aperiodic modes, other mechanisms appeared, such as ejection from the mixing layer, trapping by a growing vortex or merging vortices. The analysis of the local velocity field, the vortices’ shape, the spatial distribution of the forces and the ion flux components explained these trajectories.

## 1. Introduction

Ion exchange membranes play an important role for a wide range of engineering applications [1]. These applications can be divided in two main groups: separation processes and energy production and conversion. The main processes gathered in the separation field are electrodialysis (water desalination and salt pre-concentration), diffusion dialysis (acid and base recovery from industrial waste water), bipolar membrane electrodialysis (production of acids and bases), continuous electro-deionization (production of ultrapure water), and capacitive deionization (water desalination and water softening). Energy production and storage include reverse electrodialysis, fuel cells and redox flow batteries [2,3]. The recent development of microscale devices, such as microfuel cells, lab-on-a-chip biomolecule sensing and micropumps, use electro-kinetic phenomena in the presence of ion exchange membranes [4,5,6]. These membranes are mainly polymeric matrices composed of backbones on which cationic or anionic groups are attached. Owing to the key role of the ion exchange membrane, the role of the functional groups, polymer architecture and chemical procedure of functionalization have been extensively studied and have led to a very wide variety of membranes [1,7]. In the case of microscale devices, a new class of membrane seems to be promising. The structure of these materials is inspired by biological processes, such as enzymatic catalysis or transport regulation of species through membrane proteins [8,9,10,11].

Electro-convective instability involving an ion exchange membrane was predicted for the first time by Rubinstein and Zaltzman [12,13,14,15,16]. The authors showed by means of an asymptotic expansion that the deformation of the non-equilibrium electric double layer (EDL) induces a longitudinal force (along the membrane) initiating fluid motion [13,14]. This new kind of instability, confirmed by experimental visualizations [17,18,19,20,21,22,23], enhances the ion flux through the membrane system. This phenomenon is all the more important because electro-convective instability occurs at the microscale as it is not bound to the Reynolds number but to the non-linear coupling between the ionic charge density and the electric field.

**Figure 1:**
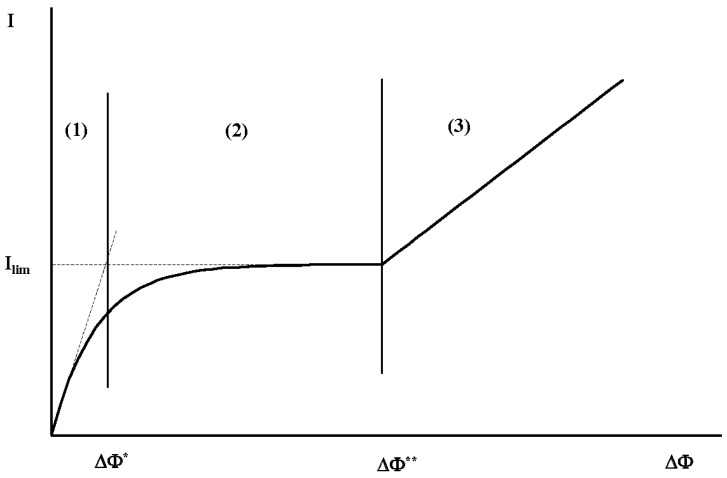
Schematic drawing of the current density (*I*) vs. potential drop (ΔΦ) over a membrane system. The regions (1), (2) and (3) localize the Ohmic regime, the limiting current regime and the overlimiting current regime respectively.

Three regimes are commonly observed in a current–voltage response when a potential drop (∆Φ) is applied normal to the membrane system (see Figure 1). For a small potential drop (region (1)), the ionic conductivity is constant (Ohmic regime). The polarization layer constitutes an electroneutral diffusion layer, over which the concentration gradient is constant, and a quasi-equilibrium EDL. The concentration gradient increases with ∆Φ, which means that the width of the diffusion layer decreases. It follows that beyond a first potential threshold ∆Φ* (region (2)), the conductivity cancels and the current density reaches a plateau (limiting current regime) [24,25,26]. A new layer, called the extended space charge (ESC) region, appears between the diffusion layer and the quasi-equilibrium EDL [12]. The ESC region and the quasi-equilibrium EDL form the non-equilibrium EDL. In the ESC region, the electro-neutrality is not fulfilled. The ESC width increases with ∆Φ. Beyond a second threshold ∆Φ** (region (3)), the non-equilibrium EDL becomes unstable. A longitudinal electric charge gradient, located in this layer, induces an electric and a pressure force along the membrane surface. The enhanced ionic transport leads to an increase in current density [27,28,29,30]. This last regime is called the over-limiting current regime. The diffusion layer is moved away from the membrane and a new layer, called the mixing layer, takes place between the diffusion one and the non-equilibrium EDL. The width of the mixing layer is much higher than the vortex size owing to a plume of small ion concentration in the region of the rising fluid [21,28,31].

Direct numerical simulations are necessary to reach a complete understanding of the electro-kinetic instability mechanism. With this aim, the Navier–Stokes equation, coupled with the Poisson–Nernst–Planck systems, has been solved in previous works for the case of one or two membranes immersed in a stationary reservoir. In this context, instability modes, transition to chaotic motion, 3D electro-convective pattern, bifurcation, non-ideal selectivity and chaotic mixing were investigated [28,31,32,33,34,35,36,37]. Recently, the influence of surface heterogeneity on the electro-instability has also been investigated by direct simulations and experimental visualizations [38,39]. In particular, the authors [38] have studied how the electric field, bended by an array of charged P2VP microgels coated on Nafion membranes, enhances the ionic transfer.

Few studies have been published in the case of electro-kinetic instabilities in the presence of an imposed inflow. Kwak et al. [18] found a scaling law governing the vortex thickness. The authors also observed that the vortex advection velocity is dependent on the imposed mean velocity. In a 3D approach, experiments and simulations reveal parallel helical vortices and a merging process along the channel [40]. Nikonenko’s group [28,41] described the instability mode appearing successively during a linear potential sweep. The authors observed first a periodic mode with a time period about 0.9 s, when ∆Φ is close to the instability threshold. For a higher potential drop, they observed regularly spaced clusters of counter-rotating vortices. They noticed that vortices can emerge in these clusters. This research group also analyzed the width of different zones of the diffusion layer as a function of the current density. In particular, they found an agreement between experiments and simulations in spite of the difference of inlet concentration.

Despite the quality of the published results, the mechanisms controlling the instability modes remain only partially understood. The goal of the current work is to intend to explain some processes like vortex dissociation/association, counter-clockwise vortex birth and also time evolution of the cluster structure by considering the spatial distribution of the electric and pressure force. As in previous publications [28,33,40,41], the fluid flow is visualized by means of streamlines computed at a given time. Therefore, the time evolution of the vortex layer is a snapshot series of these streamlines named ‘static’ (SSL) in the article. However, considering the unsteady fluid flow, the fully Lagrangian approach provides access to the real hydrodynamics. This is visualized by trajectories called ‘dynamic’ (DSL) streamlines in the present work. So, in a second step, the close relation and complementarity between these ‘dynamic’ streamlines and the vortices computed with the SSL are clearly identified and analyzed with the spatial distribution of force and kinetic energy. This gives a new insight into the role of the vortices in the fluid motion and of the relation between the diffusion layer and the real instability layer. This approach is also extended to ion transfer in the periodic mode. Finally, the results are summarized in the conclusion.

## 2. Theory

### 2.1. Model and Governing Equations

**Figure 2:**
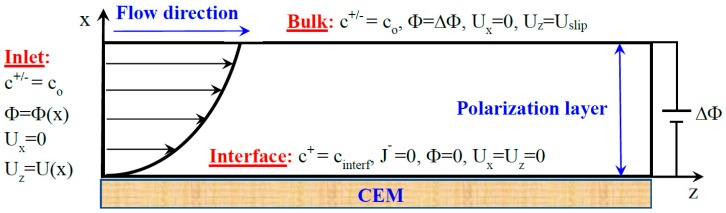
Schematic representation of the numerical model: an electric potential drop ΔΦ is imposed over the width of the electrolyte layer (polarization layer) lying between a bulk, containing an electrolyte solution of concentration $c_{o}$ and a cation exchange membrane at the surface of which the cation concentration $c_{\mathrm{interf}}^{+}$ and a non-flux of co-ions are imposed. A Poiseuille flow is imposed at the inlet.

**Figure 3:**
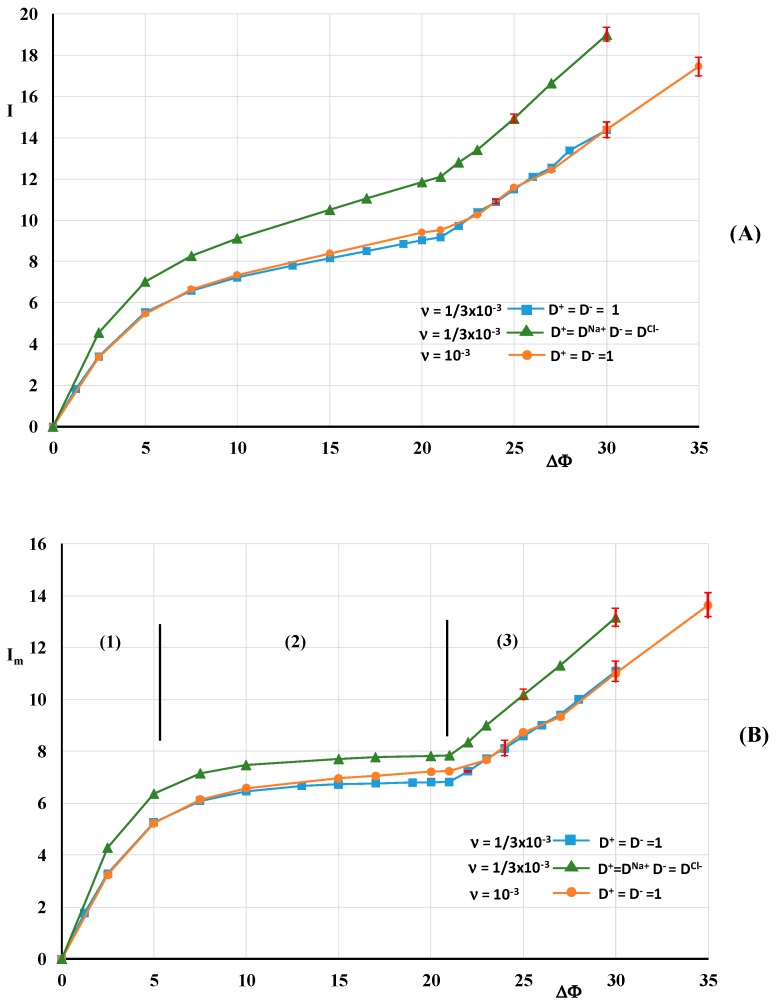
Current density vs. potential drop. (**A**): mean current density through the domain (*I*); (**B**): current density at the membrane surface (*I*_m_). The red vertical bars represent the variance of the current density signal.

The elementary electrodialytic cell consists of a channel between two parallel ion exchange membranes. The electrolyte solution enters one end of the channel and exits from the other. The transverse electric potential drop, imposed at the two outer sides of the membranes, induces two polarization layers at their inner sides [41]. In the current work (Figure 2), the membrane system consists of one polarization layer close to a cation exchange membrane of length *L_z_*. The bulk is located at *L_x_* from the membrane. At the inlet (*z* = 0), the ionic concentration is $c_{o}^{}$, a constant transverse potential gradient $\Delta \Phi /L_{x}$ is imposed and a part of the Poiseuille profile is prescribed depending on the distance between the two membranes $L_{m}\left(>L_{x}\right)$. At the membrane surface (x=0), the cation concentration is $c_{\mathrm{interf}}^{+}$, the anion flux and the electric potential are zero. These two last conditions mean that the membrane is ideally selective and its electrical resistivity is zero as well. At the bulk limit ($x=L_{x}$), the potential and the ion concentration are imposed constant and equal to ΔΦ and $c_{o}^{}$, respectively. The fluid velocity is longitudinal and equal to *U*_slip_. Figure 3 displays a snapshot of cationic profiles and streamlines for two values of ∆Φ in the case of the monovalent electrolyte (Na^+^, Cl^−^).

The incompressible fluid motion is described by the Stokes equation (Equation (1)) coupled with the continuity equation (Equation (2)). The ionic transport is governed by the Nernst–Planck equation (Equation (3)) and the electric potential is related to the local ionic concentration by the Poisson equation (Equation (4)):

(1) $$0=-\vec{\nabla }P+\mu \Delta \vec{U}-\rho_{f}\left(z^{+}c^{+}+z^{-}c^{-}\right)\vec{\nabla }\Phi$$

(2) $$\vec{\nabla }\cdot \vec{U}=0$$

(3) $$\frac{d}{dt}c^{\pm }=\frac{\partial }{\partial t}c^{\pm }+\vec{U}\cdot \vec{\nabla }c^{\pm }=D^{\pm }\vec{\nabla }\cdot \left(\vec{\nabla }c^{\pm }+\frac{z^{\pm }c^{\pm }}{k_{B}T}\vec{\nabla }\Phi \right)$$

(4) $$-\varepsilon_{0}\varepsilon_{r}\Delta \Phi =Q=z^{+}c^{+}+z^{-}c^{-}$$

Here *P* is the pressure, *µ* is the dynamic viscosity, $\vec{U}$ is the fluid velocity, $\rho_{f}$ is the fluid density, $z^{\pm }$ is the charge of the cation and the anion, $c^{\pm }$ is the concentration of the cation and of the anion, Φ is the electric potential, *t* is the time, $D^{\pm }$ is the diffusion coefficient of the cation and of the anion, *Q* is the electric charge density, $\varepsilon_{o}$ and $\varepsilon_{r}$ are the permittivity of the vacuum and the relative permittivity of the solution, respectively, and $k_{B}$ and *T* are the Boltzmann constant and the temperature, respectively.

These equations are scaled as in Druzgalski et al. [31]. The characteristic parameters are the transverse length $L_{x}^{}$, the diffusional time $t_{diff}=L_{x}^{2}/D_{ref}$, the diffusional velocity $U_{diff}=D_{ref}/L_{x}$, the thermodynamic potential $\Phi_{T}=k_{B}T/e$, the ‘osmotic’ pressure $P_{o}=\mu D_{ref}/L_{x}^{2}$ and the bulk concentration *c*_o_. Here *e* is the elementary charge, $D_{ref}$ is a reference diffusion coefficient whose value is arbitrary. For a symmetric univalent electrolyte, Equations (1)–(4), in their dimensionless form, become:

(5) $$0=-\vec{\nabla }P+\Delta \vec{U}-\frac{Pe}{2\nu^{2}}\left(c^{+}-c^{-}\right)\vec{\nabla }\Phi$$

(6) $$\vec{\nabla }\cdot \vec{U}=0$$

(7) $$\frac{d}{dt}c^{\pm }=\frac{\partial }{\partial t}c^{\pm }+\vec{U}\cdot \vec{\nabla }c^{\pm }=D^{\pm }\vec{\nabla }\cdot \left(\vec{\nabla }c^{\pm }\pm c^{\pm }\vec{\nabla }\Phi \right)$$

(8) $$-2\nu^{2}\Delta \Phi =Q=c^{+}-c^{-}$$

Here Pe and ν are the Peclet number and the dimensionless Debye length, respectively:

(9) $$Pe=\frac{\varepsilon_{o}\varepsilon_{r}}{\mu D_{ref}}{\left(\frac{k_{B}T}{e}\right)}^{2}$$

(10) $$\nu =\frac{\lambda_{d}}{L_{x}}\;\mathrm{with}\;\lambda_{d}={\left(\frac{\varepsilon_{o}\varepsilon_{r}k_{B}T}{2e^{2}c_{o}}\right)}^{1/2}$$

For simple ions, the diffusion coefficient is roughly $10^{-9}$ m^2^/s. Hence, this value will be used to define $D_{ref}$ and the value of Pe is imposed equal to 0.5. Therefore, if $L_{x}=0.1\;\mathrm{mm}$ and $\nu =10^{-3}$, then $\lambda_{d}=100\;\mathrm{nm}$, $c_{o}=10^{-5}\;M$ (T=300 K) and the diffusional time is 10 s. In what follows, the dimensionless variables will be used. Therefore, the value of dimensionless transverse length $L_{x}$ and of the dimensionless bulk concentration $c_{o}$ are equal to 1.

The boundary conditions used at the fluid/membrane interface are the no-slip condition, and the imposed value of the cationic concentration $c_{\mathrm{interf}}^{+}$ is assumed to be equal to $c_{o}$. At the inlet, the imposed velocity profile has the following expression:

(11) $$U_{z}=6\langle U_{z}\rangle \frac{x}{L_{m}}\left(\frac{x}{L_{m}}-1\right)$$

In this expression, $\langle U_{z}\rangle$ is the longitudinal velocity averaged over the distance between the two membranes and x ranges from 0 to $L_{x}$. Therefore, the slip velocity imposed at the bulk boundary is $U_{slip}=U_{z}\left(z=0,x=L_{x}\right)=6\langle U_{z}\rangle L_{x}/L_{m}\left(L_{x}/L_{m}-1\right)$. At the outlet ($z=L_{z}$), the equality of the velocity, of the ion concentration and of the electric potential are imposed on the two sides of the boundary [18]. As the Stokes equation is solved with the finite volume method, the pressure is linearly extrapolated at all the domain boundaries.

At the membrane surface, the no-slip boundary condition is usually applied. However, the slip condition can be justified by the hydrophobic property of membranes. The velocity slip is modeled by the Navier equation, which is characterized by the slip length. The value of this parameter depends on the degree of hydrophobicity and heterogeneity. It is admitted that it is lower than 100 nm for a homogeneous surface and may reach a few micrometers for a grooved surface (superhydrophobicity) [42,43]. Experiments on cation exchange membranes have shown that surface modification by hydrophobization enhances the electro-convection [44,45]. The instability threshold is displaced to lower values of ∆Φ, and at a given value of ∆Φ, the current density increases. The consequence is that water splitting and salt precipitation are reduced. Numerical simulations [41,46] confirm the experimental observations. Shelistov et al. [46] have shown that moderate hydrophobicity increases the critical wavelength and the maximal growth rate, and the unsteady mode appears at lower values of ∆Φ as the hydrophobicity increases.

In this study, $L_{z}=6L_{x}$ and $L_{m}=\left(10/3\right)L_{x}$, $\langle U_{z}\rangle =240$ (8 mm/s), and ΔΦ varies up to 35 (0.9 V). Two values of ν were used ($10^{-3}$ and ${1/3\times 10}^{-3}$) and two cases were also considered for $D^{\pm }$ ($D^{+}=D^{-}=1$ and $D^{+}=D^{Na+}=1.33$, $D^{-}=D^{Cl-}=2.06$). In the following, the ionic flux and the current density are defined in their dimensionless form:

(12) $$\vec{J}{}^{\pm }=c^{\pm }\vec{U}-D^{\pm }\left(\vec{\nabla }c^{\pm }\pm c^{\pm }\vec{\nabla }\Phi \right)\;\mathrm{and}\;I={\langle {\left(\vec{I}\right)}_{x}\rangle }_{\Omega }=\frac{1}{A}\int_{\Omega }\left({\left(\vec{J}{}^{+}\right)}_{x}-{\left(\vec{J}{}^{-}\right)}_{x}\right)dA$$

In this equation, A is the area of the computational domain Ω.

### 2.2. Numerical Method

#### 2.2.1. Stokes and Poisson–Nernst–Planck Equations

The same discretization method and structured grid refinement as the previous article [37] were used. The number of nodes is 180 and 384 in the transverse and longitudinal direction, respectively. In the transverse direction, the common ratio of the geometric series is 0.0234. In the longitudinal direction, the refinement threshold is 2.5 Equations (5)–(8) are integrated in time with a time step δt of $10^{-4}$.

#### 2.2.2. Streamlines

**Figure 4:**
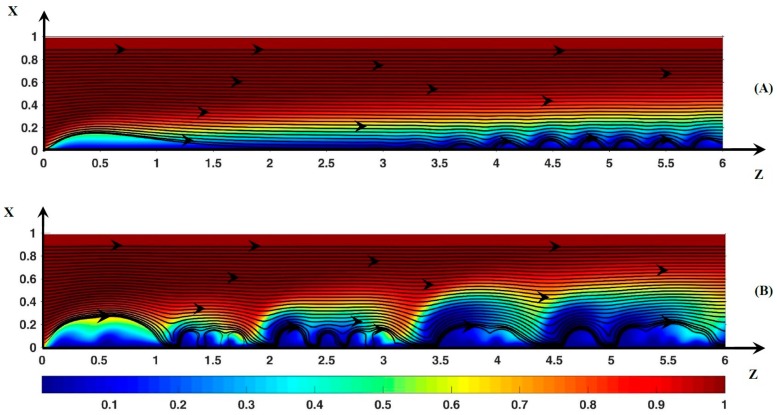
Color plot of the cationic concentration. (**A**): ΔΦ = 22, (**B**): ΔΦ = 30. The black lines represent the static streamlines.

In References [18,28,30,31,32,40,41] the hydrodynamic streamlines were computed with the velocity field at a given time. In the present work, these streamlines are named static streamlines (SSL) and are computed with Matlab. In Section 3.2, the instability structure is analyzed with the SSL. They are visualized in black in Figure 4 for example. In Section 3.3, the real trajectories of the fluid elements and ions are computed with the numerical method described below. These trajectories will be named dynamic streamlines (DSL) owing to the use of the unsteady fluid and ion transfer. The trajectories are displayed in red in Section 3.3. 

Two kinds of dynamic streamlines are computed in this work: the hydrodynamic and the ionic one. As regards to the ions, their local velocity is computed by means of the ratio between the flux density and the concentration: ${\vec{J}}^{\pm }/c^{\pm }$. As the velocity field and the spatial distribution of concentrations and potential are unsteady, the time integration is performed by means of the fourth order Runge Kutta method.

Consider an elementary volume of fluid located at the point $\left(z_{o},x_{o}\right)$ at time $t_{o}$. Four Eulerian values of velocity are needed to compute the Lagrangian one:

$$\vec{U}{}_{1}=\vec{U}\left(z_{o},\;x_{o},\;t_{o}\right)$$

$$\vec{U}{}_{2}=\vec{U}\left(z_{o}+{\left(\vec{U}{}_{1}\right)}_{z}\times \delta t/2,\;x_{o}+{\left(\vec{U}{}_{1}\right)}_{x}\times \delta t/2,\;t_{o}+\delta t/2\right)$$

(13) $$\vec{U}{}_{3}=\vec{U}\left(z_{o}+{\left(\vec{U}{}_{2}\right)}_{z}\times \delta t/2,\;x_{o}+{\left(\vec{U}{}_{2}\right)}_{x}\times \delta t/2,\;t_{o}+\delta t/2\right)$$

$$\vec{U}{}_{4}=\vec{U}\left(z_{o}+{\left(\vec{U}{}_{3}\right)}_{z}\times \delta t,\;x_{o}+{\left(\vec{U}{}_{3}\right)}_{x}\times \delta t,\;t_{o}+\delta t\right)$$

The Eulerian fluid velocity $\vec{U}{}_{1}$ at a point $\left(z_{o},x_{o}\right)$ at time $t_{o}$ is computed by a spatial interpolation with an upstream biquadratic polynomial. $\vec{U}{}_{2}$ is computed in two steps. First a quadratic time interpolation of the nine velocities, used for the spatial interpolation, must be carried out because ${\vec{U}}_{{}_{2}}$ is determined at the intermediate time $t_{o}+\delta t/2$. Then the spatial upstream biquadratic interpolation is performed with the velocities interpolated in time. The time interpolation is performed with the velocity fields at time $t_{o}-\delta t$, $t_{o}$, and $t_{o}+\delta t$. The velocities ${\vec{U}}_{{}_{3}}$ and ${\vec{U}}_{{}_{4}}$ are computed in the same way as ${\vec{U}}_{{}_{2}}$ and ${\vec{U}}_{{}_{1}}$, respectively. The computation of the ionic Lagrangian velocity is carried out in the same manner with the three flux contributions (${\vec{J}}_{Co}^{\pm }=c^{\pm }\vec{U}$,${\vec{J}}_{Fi}^{\pm }=-D^{\pm }\vec{\nabla }c^{\pm }$,${\vec{J}}_{Em}^{\pm }=\mp D^{\pm }c^{\pm }\vec{\nabla }\Phi$) and with the ion concentration; here *Co*, *Fi* and *E_m_* mean convective, Fickian and electro-migration contribution, respectively. The interpolated flux is divided by the interpolated concentration to determine the four ${\vec{U}}^{\pm }$.

## 3. Numerical Results

### 3.1. Comparison with Published Results

To validate the numerical method, the results were compared to those published in References [18,28,41]. To my knowledge, no analytical solution is available in the literature. Several analyses of the polarization layer structure in an over-limiting regime have been carried out by assuming 1D ion transfer at steady state (see Zaltzman et al. [14] and references therein). With these assumptions, the electric field is a solution of the 2nd kind Painlevé equation which does not admit a simple analytical solution.

Figure 3 shows the mean current density through the boundary layer (*I*) and through the membrane surface (*I_m_*) as a function of the potential drop. Three series of computations were carried out. Each point of the curves corresponds to the value of the current density averaged in time at a prescribed potential drop. In the Ohmic and limiting regimes, the potential drop increases by a step of 2.5 until it reaches a value of 21. In the over-limiting regime, a first series is carried out with $D^{\pm }=1$, $\nu =1/3\times 10^{-3}$. Beyond the value of 22, the initial condition is the unsteady solution at the previous value of ΔΦ. Concerning the other series, the initial condition at a given potential drop is the unsteady solution of the first series with the same value of ΔΦ in order to reduce the transitory period. The series 1 and 2 ($D^{+}=D^{Na+}$, $D^{-}=D^{Cl-}$, $\nu =1/3\times 10^{-3}$) will be used for the comparison with published numerical results and theoretical ones. The 3rd series is computed with $D^{\pm }=1$ and $\nu =10^{-3}$. The time average of the current density is computed after a stabilization period of the current signal. By stabilization it is not meant that steady state is recovered. The transitory period can take several hundred time steps, especially after a jump of the potential. The current density is averaged over one to two thousand time steps. In Section 3.2 and Section 3.3, the instability structure and the dynamic streamlines are analyzed after the transitory period.

These series show that the over-limiting threshold values lie between ΔΦ=21 and 22 (0.57 V). This value, slightly sensitive to the diffusion coefficient and the length ratio ν, is higher than the value found in the case of periodic conditions [31,37]. In Nikonenko et al. [28], an expression of the local limiting current density, in the case of a laminar flow between two plates, was proposed. Averaged over the domain length, the limiting current density $I_{\lim}$ has the following expression (Equation (17.56) in Newmann et al. [47]):

(14) $$I_{\lim}=\frac{2}{L_{x}}\left[1.47{\left(\frac{L_{x}^{2}\langle U_{z}\rangle }{L_{z}D}\right)}^{1/3}\right]$$

where $D=2D^{+}D^{-}/\left(D^{+}+D^{-}\right)$. The expression (14) is equivalent to Equation (3b) in Nikonenko et al. [28] because the cation transfer numbers $T_{1}$ and $t_{1}$ are equal to 1 and 0.5, respectively. In Equation (14) $L_{x}$ is used instead of $L_{m}$ because the potential drop is imposed over $L_{x}$. In the same idea, $\langle U_{z}\rangle$ is the inlet velocity averaged over the computational domain width. This relation gives a value of 9 and 12.4 for the series 1 and 2, respectively. Therefore, it seems from Figure 3 that *I* is the relevant quantity in the present model.

**Figure 5:**
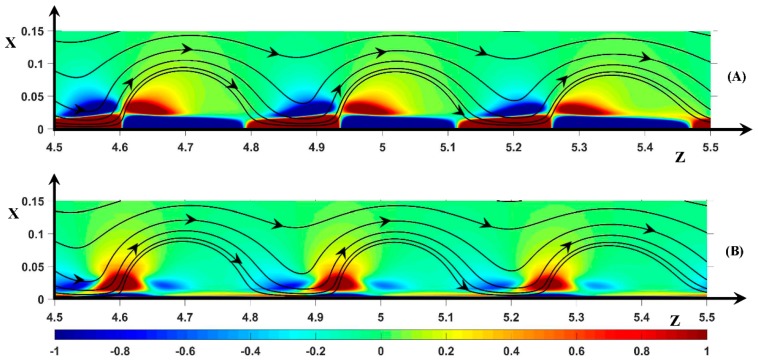
Color plot of: (**A**) ${\left(\vec{F}{}^{Tot}\right)}_{z}$; (**B**) ${\left(\vec{F}{}^{Tot}\right)}_{x}$. Black lines: static streamlines starting from the inlet. ΔΦ = 22, $\nu =1/3\times 10^{-3}$, $D^{\pm }=1$.

Let us compare the cation profiles obtained in the present work and those published in References [28,41]. The authors investigated the ion transfer between an anion exchange membrane and a cation one. In Urtenov et al. [41], the two membranes were non-ideal, i.e., co-ions could cross the membranes. The potential drop is imposed at the outer membrane surfaces. In this publication, Figure 5 shows the static streamlines and the map of the cation concentration with the parameter values $L_{x}=L_{m}=1\;\;\mathrm{mm}$, $L_{z}=2\;\;\mathrm{mm}$, $\nu =10^{-4}$, $\langle U_{z}\rangle =8\times 10^{-4}\;{\mathrm{ms}}^{-1}$, $D^{+/-}=D^{Na+/Cl-}$. Therefore, if a part of the domain is modeled, i.e., $L_{x}=0.3\;\mathrm{mm}$, one obtains $\nu =1/3\times 10^{-3}$, which corresponds to series 2 if $D^{+/-}=D^{Na+/Cl-}$. However, the value of the potential threshold is close to 1 V instead of 0.57 V. Two reasons may explain this difference in value: (1) The potential gradient is located mainly in high resistance regions, i.e., where the ion concentration is low. So, the transverse gradient takes place in the two polarization layers [28,41]. Assuming the problem is symmetric, the potential drop through the cationic polarization layer would be equal to about half of the overall potential drop. (2) The membranes have a non-zero electrical resistivity and the potential drop is imposed outside the electro-dialytic cell. These two conditions lead to an increase in the potential threshold. However, it also seems that the potential threshold depends on *L_m_* [28,41]. At the threshold, the authors [41] found that the outlet cation concentration was equal to 0.6 at $x=x_{c^{+}=0.6}=L_{m}/10$ (100 µm) from the cation exchange membrane, which corresponds to $L_{x}/3$ in the present work at ΔΦ=22 (see Figure 4A). Their values ΔΦ=1.8 V, $c^{+}=0.6$ at $x_{c^{+}=0.6}=L_{m}/7$ (140 µm) correspond to ΔΦ=30=1.4×21.5, $c^{+}=0.6$ at $x_{c^{+}=0.6}=0.5L_{x}$ (150 µm) found here. The counterion concentration color map shown in Figure A2 (Nikonenko et al. [28]) come from simulations carried out with the same type of membrane cell ($L_{x}=L_{m}=0.5\;\mathrm{mm}$, $L_{z}=2\;\mathrm{mm}$, $\langle U_{z}\rangle =8\times 10^{-4}\;{\mathrm{ms}}^{-1}$, $D^{+/-}=D^{Na^{+}/Cl^{-}}$) and with several inlet ion concentrations. Two cases may be used for comparison: $\nu =2\times 10^{-4}$ (Figure A2a) and $\nu =6\times 10^{-5}$ (Figure A2b). In these Figures $\Delta \Phi =1.43\Delta \Phi^{**}$ with $\Delta \Phi^{**}=0.7V$. In the present work, $L_{m}=0.5\;\mathrm{mm}$ and $\Delta \Phi =1.43\Delta \Phi^{**}$ correspond to $L_{x}=0.15\;\mathrm{mm}$ and ∆Φ = 31, respectively. In Figure A2a,b, $c^{+}/c_{o}=0.6$ at $x_{c^{+}=0.6}=0.6L_{x}$ (90 µm), which is close to $x_{c^{+}=0.6}=0.5L_{x}$ (75 µm) obtained with $\nu =1/3\times 10^{-3}$ (Figure 4B). The color bar range in terms of dimensionless concentration is assumed to be identical to the range in Figure 5 (Urtenov et al. [40]). The values of $x_{c^{+}=0.6}$ are given in Table 1.

Kwak et al. [18] found a linear correlation between the vortex size $\delta_{v}$ and the potential drop ∆Φ_v_ over $\delta_{v}$: $\delta_{v}/L_{m}=a{\left(\Delta \Phi_{v}^{2}/\langle U_{z}\rangle \right)}^{1/3}+b$ where $\langle U_{z}\rangle$ is the mean inlet velocity (see Table 2). With their numerical model, Urtenov et al. [41] verified this linear behavior. In the present work, as ΔΦ increases from 23 to 30 (Figure 4), at the outlet $\Delta \Phi_{v}$ and $\delta_{v}/L_{m}$ increase from 19.4 to 25.7 and from 0.04 to 0.07, respectively. If the velocity is averaged over $L_{x}$, the values of a and b are very close to those found by Urtenov et al. [41] (Table 2). Similar values are found if the velocity is averaged over $L_{m}$ (a = 0.0215, b = 0.107).

### 3.2. Hydrodynamic Instability

The Nikonenko research group has mainly investigated the structure of the polarization layer. However, the instability modes are briefly studied, and the cluster dynamics and structure are not addressed. On the contrary, in this section, the key role of the force spot upstream of the vortex front in the vortex shape and in the birth of counter-clockwise vortices and clusters is shown as an example. In Section 3.3, the role of SSL bending, induced by the layer ${\left(\vec{F}{}^{Tot}\right)}_{{}_{z}}<0$ at the vortex rear, in the fluid flux through the outer edge of the unsteady vortex layer will be also pointed out. The investigation is performed with $D^{\pm }=1$ and $\nu =1/310^{-3}$. Another mode of instability found with $D^{\pm }=1$ and $\nu =10^{-3}$ is briefly discussed in the Appendix A.

#### 3.2.1. Periodic Mode Flow (ΔΦ=22)

Before going further, the main results described in the previous article [37] about the spatial distribution of the electric and pressure forces and of the fluid kinetic energy are recalled briefly. In forced flow, the periodic mode close to the instability threshold is very similar to the marginal instability because the rolls are stable during their travel to the outlet. In periodic systems, the steady instability is characterized by two counter-rotating vortices. The non-equilibrium EDL has a minimum width in the downward flow region and presents a hump at the upward one. The hump extends between the two vortex centers. *Q* decreases mainly in the hump because its transverse mean value remains constant. It is recalled that the non-equilibrium EDL gathers the ESC region and the quasi-equilibrium EDL. Two force layers along the membrane induce a longitudinal flow. In the first layer placed in the quasi-equilibrium EDL, the pressure force is dominant, whereas the electric one is dominant in the second layer located around the local maximum of electric charge density (*Q*_max_) in the ESC region. Two spots of kinetic energy are located in the second layer and close to the ESC hump. A third spot is located between the two vortices in the upward region due to the convergence of the fluid streamlines. The pressure and potential disequilibrium on both sides of the vortices is explained first by the transverse profile of charge density, which controls the variation of the potential gradient, and also by the equilibrium between the electric force and the pressure one in the transverse direction leading to the quadratic dependence of the pressure on the transverse potential gradient.

Figure 5 displays the vortices (SSL) and the color map of the two components of the total force $\left(\vec{F}{}^{Tot}\right)$, which is the sum of the electric $\left(\vec{F}{}^{El}\right)$ and pressure force $\left(\vec{F}{}^{Pr}\right)$. All vortices rotate clockwise. The region of upward fluid motion defines the front of the vortex. Inversely, the region of downward fluid motion defines the rear of the vortex. The space between two successive vortices, i.e., the inter-vortex region, replaces the counter-clockwise vortex in the periodic model. The circumstances in which a counter-clockwise vortex occurs this space will be discussed. The minimum width of the non-equilibrium EDL is located at the rear of the vortex and the hump takes place around the vortex front (upward flow). The two spots of force (Figure 5A) are equivalent to the spots shown in Reference [37] but with a much higher intensity. In these spots, the pressure force is dominant.

A layer and a spot of the same force sign, located at two different transverse positions, may be linked as shown in Figure 5A. This means that a continuous path of the same force sign may go through the front or the rear of the vortex. This controls the inclination of the static streamlines. For this reason, the streamlines are oriented downstream of the front. When there is no link, the front is vertical. At the rear, in the vortex located at *z* ≈ 5.3 for example, the spot ${\left(\vec{F}{}^{Tot}\right)}_{{}_{z}}>0$ is linked to the layer of positive sign, whereas, in the upstream vortices rear (*z* ≈ 5 and *z* ≈ 4.7), the pair spot/layer ${\left(\vec{F}{}^{Tot}\right)}_{{}_{z}}<0$ are linked. This explains the small difference in orientation of the streamlines at the rear of the two vortices and the small deviation from the exact periodicity. Below, the effect of these spot/layer links on the form of the vortices and their influence on the vortex dynamics will be more clearly shown and described.

Figure 5B shows the transverse component of the total force ${\left(\vec{F}{}^{Tot}\right)}_{{}_{x}}$. ${\left(\vec{F}{}^{El}\right)}_{{}_{x}}$ is always negative as a consequence of the positive sign of *Q*. The transverse pressure force is opposed to the electric one in the front region, whereas it is negative in the rear region. Three spots are located around the vortex front. At the vortex front, the pressure force dominates (${\left(\vec{F}{}^{Tot}\right)}_{{}_{x}}>0$). The spot is located inside the ESC region. On both sides of the front, the electric force is dominant (${\left(\vec{F}{}^{Tot}\right)}_{{}_{x}}<0$) suppressing the upward fluid motion. The bottom of these two spots are located at the outer edge of the ESC region [37].

**Figure 6:**
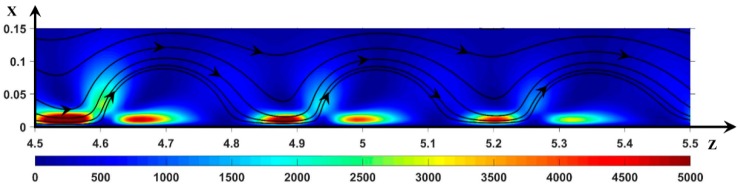
Color plot of the hydrodynamic kinetic energy *E*_c_. Black lines: static streamlines starting from the inlet. ΔΦ = 22, $\nu =1/3\times 10^{-3}$, $D^{\pm }=1$.

In Figure 6, the fluid kinetic energy ($E_{c}$) also presents three spots of high magnitude. However, the kinetic energy at the front is much smaller than when there is no forced flow [37], and it induces a smaller roll size. This also induces a periodic trajectory of the anions (see Section 3.3.3). A disequilibrium of $E_{c}$ is also visible on both sides of the front. As when there is no forced flow, the two $E_{c}$ spots are located at the transverse position of *Q*_max_: the fluid is accelerated in the longitudinal direction by the electric force and reaches a maximum kinetic energy when *Q*_max_ and the ESC width begin to decrease and increase, respectively.

**Figure 7:**
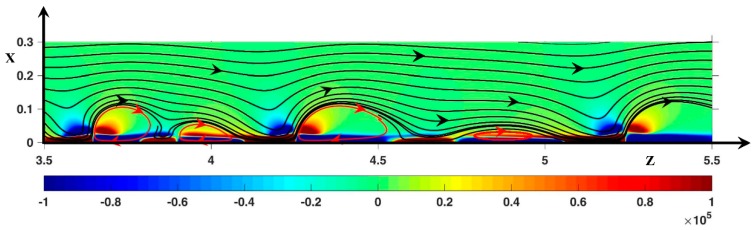
Color plot of ${\left(\vec{F}{}^{Tot}\right)}_{z}$. Black lines: static streamlines. ΔΦ = 23, $\nu =1/3\times 10^{-3}$, $D^{\pm }=1$.

**Figure 8:**
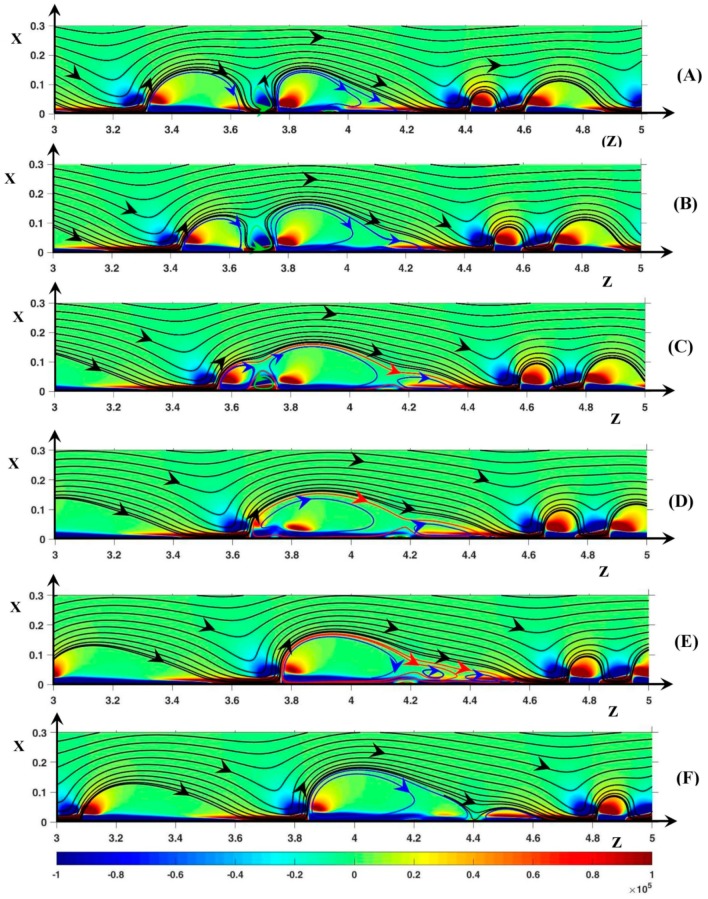
Temporal evolution of the cluster structure. (**A**) *t*’ = 0; (**B**) *t*’ = 20; (**C**) *t*’ =40; (**D**) *t*’ = 60: (**E**) *t*’ = 80; (**F**) *t*’ = 100. Color plot: ${\left(\vec{F}{}^{Tot}\right)}_{z}$. Black lines: static streamlines starting from the inlet. Blue streamlines: clockwise rotating rolls, green streamlines: counter-clockwise rotating rolls, red streamlines: cluster envelope. ΔΦ = 27, $\nu =1/3\times 10^{-3}$, $D^{\pm }=1$.

Contrary to [28,41], another periodic mode is also observed at ∆Φ = 23. The period of the vortex structure is composed of a vortex pair formed from a large and a small vortex (Figure 7). The vortex pair size is seen to increase with time. Two consecutive vortices begin to form a pair when the upstream vortex (V1) reaches the position z=3.5. The pair formation consists of three steps. During the first one, the downward vortex (V2) comes closer to the downstream pair so that its size decreases. At the two ends of V2, the streamlines are vertical close to the membrane because the negative layer and the negative spots ${\left(\vec{F}{}^{Tot}\right)}_{{}_{z}}$ are linked at these ends (for an example, see a similar situation at *z* = 4.7 in Figure 8D). In the second step, when V2 reaches its minimum size, V1 begins to move closer to V2. The negative layer and the negative spot ${\left(\vec{F}{}^{Tot}\right)}_{{}_{z}}$ get closer at the rear of V1, whose size decreases during its displacement. In the third step, the negative spot ${\left(\vec{F}{}^{Tot}\right)}_{{}_{z}}$ between V1 and V2 flattens and the two layers ${\left(\vec{F}{}^{Tot}\right)}_{{}_{z}}<0$ at the rear of V1 join leading to vertical streamlines near the interface. At the same time, at the rear of V2, the positive spot and the positive layer ${\left(\vec{F}{}^{Tot}\right)}_{{}_{z}}$ also join. The length of V2 increases and its height decreases accordingly. Figure 7 shows the third and final step.

#### 3.2.2. Non-Periodic Mode Flow (∆Φ = 27)

**Figure 9:**
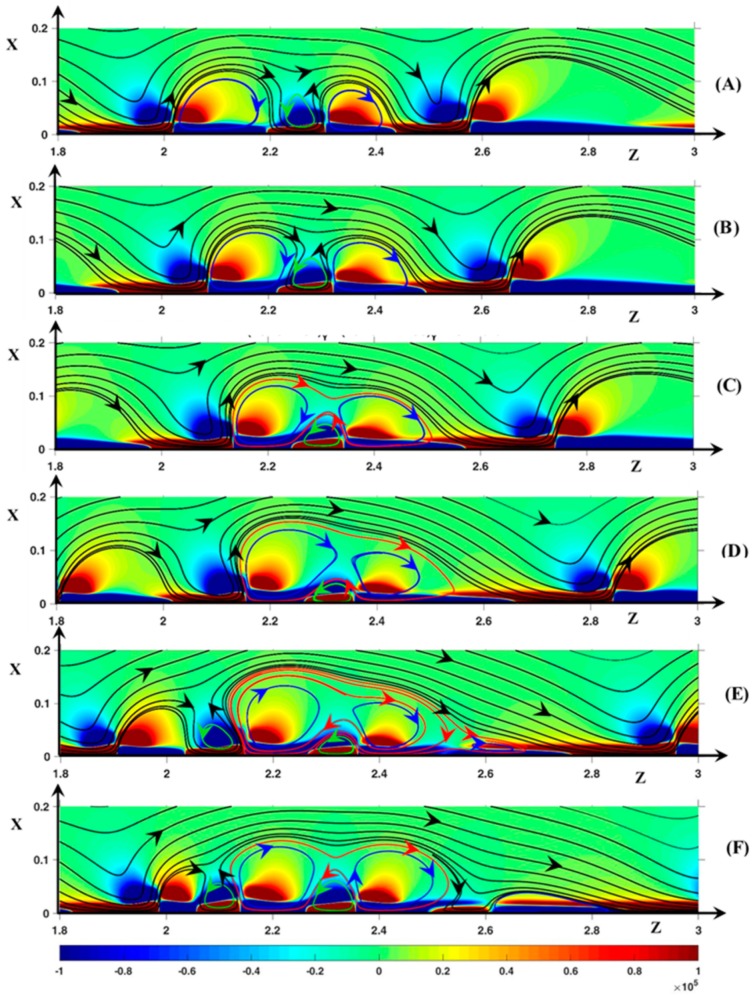
Temporal evolution of the cluster structure. (**A**) *t*’ = 0; (**B**) *t*’ = 20; (**C**) *t*’ =40; (**D**) *t*’ = 60: (**E**) *t*’ = 80; (**F**) *t*’ = 100. Color plot: ${\left(\vec{F}{}^{Tot}\right)}_{z}$. Black lines: static streamlines starting from the inlet. Blue streamlines: clockwise rotating rolls, green streamlines: counter-clockwise rotating rolls, red streamlines: cluster envelope. ΔΦ = 27, $\nu =1/3\times 10^{-3}$, $D^{\pm }=1$.

As the potential drop increases, the periodicity disappears gradually. At ∆Φ = 27 and higher, an unsteady instability mode is clearly established and the rolls associate to form clusters. The simulations with $D^{+/-}=D^{Na+/Cl-}$, ∆Φ = 27, $\nu =1/3\times 10^{-3}$, show vortex groups independent from one another as observed in Nikonenko et al. [28] and no roll exchange occurs. Inside the groups, dissociation and association are observed. With $D^{\pm }=1$, the spatial periodicity of the vortex group is not observed, and the clusters are a transitory structure. They become one vortex (Figure 8) or break into several rolls (Figure 9). It must be noticed that the definition of envelope and cluster is not the same as in Nikonenko et al. [28]. In the latter article, the envelope is the black SSL around a vortex group and a cluster is a counter-rotating vortex pair. In the current work, a cluster is a vortex group wrapped in an envelope (e.g., red SSL in Figure 8C). It does not contain counter-clockwise rolls but several clockwise ones.

In Figure 8 and Figure 9, the static streamlines (SSL) are associated with the color map of ${\left(\vec{F}{}^{Tot}\right)}_{{}_{z}}$. The blue, the green and the red SSL represent clockwise rolls, counter-clockwise rolls and the cluster envelopes, respectively. The time evolution of the cluster begins at δ*t* = 1600δ*t* (with *δt* = 10^−4^). For the sake of simplicity, this time is used as the initial one and the time is expressed in terms of *δt* in the comments (with *t*’ = *t*/*δt* − 1600).

First, let us consider Figure 8. In Figure 8A (*t*’ = 0), a counter-clockwise roll (V1/−) lies between the two clockwise vortices V1/+ and V2/+ located at *z* = 3.4 and 3.8, respectively. The signs + and − refer to the rotation direction. In Figure 8A at the rear of V2/+, the positive layer ${\left(\vec{F}{}^{Tot}\right)}_{{}_{z}}$ is the pressure force layer. It can be recalled that the negative layer ${\left(\vec{F}{}^{Tot}\right)}_{{}_{z}}$ is composed of the pressure layer next to the membrane and the electric one just above. Owing to the negative pressure force spot between V1/+ and V2/+, the black SSL at the rear of V1/+ and at the front region of V2/+ are vertical. V1/− is surrounded by the fluid film separating the vortex from V1/+, V2/+ and the membrane. V1/− rotates counter-clockwise due to the friction with the fluid film, despite the negative sign of ${\left(\vec{F}{}^{Tot}\right)}_{{}_{z}}$ at the roll bottom. That is the pressure force acts against the fluid motion at the bottom of V1/−. The inter-vortex region can be viewed as a cavity with a slip condition at the walls. This condition is induced by the fluid film.

In Figure 8B (*t*’ = 20), the volume of V1/− increases and its bottom moves to the membrane, so that it is clearly located inside the positive layer ${\left(\vec{F}{}^{Tot}\right)}_{{}_{z}}$. However, the thin fluid film continues to separate V1/− from the membrane. The vortex V1/+ front gets closer to V2/+ which does not move. The negative pressure spot ${\left(\vec{F}{}^{Tot}\right)}_{{}_{z}}$ begins to join the layer of the same sign through the front of V2/+. A new vortex appears in the tail of V2/+.

In Figure 8C (*t*’ = 40), the negative layer ${\left(\vec{F}{}^{Tot}\right)}_{{}_{z}}$ inside V1/+ connects with the negative spot ${\left(\vec{F}{}^{Tot}\right)}_{{}_{z}}$ inside V1/−. Therefore, a continuous path occurs along the membrane joining V1/+ and V2/+. This coincides with the apparition of an envelope around V1/+ and V2/+. Now, V2/+ clearly constitutes two rolls: V2a/+ (head) and V2b/+ (tail). During the merging process (Figure 8A–E), V2/+ remains at the same place, so that the distance from the downstream vortex increases. The very long non-equilibrium EDL becomes unstable and a hump of charge appears at the front of V2b/+. The SSL forming the rear of V1/+ and the front of V2/+ respectively are clearly tilted. Moving downward, V1/− gets into contact with the membrane as explained in Figure 9. However, V1/− remains isolated from V1/+ and V2/+ by the fluid film forming the cluster.

In Figure 8D (*t*’ = 60), V1/− and the front of V2/+ disappear owing to the decrease in the charge hump in the non-equilibrium EDL. The spot ${\left(\vec{F}{}^{Tot}\right)}_{{}_{z}}>0$ and the trace of the negative spot ${\left(\vec{F}{}^{Tot}\right)}_{{}_{z}}$ on both sides of the previous front of V2/+ remain. As soon as the charge hump disappears, the former spot moves upstream and joins the positive spot ${\left(\vec{F}{}^{Tot}\right)}_{{}_{z}}$ of V1/+. In Figure 8E (*t*’ = 80), V2/+, i.e., the charge hump moves downstream this time. The tail is composed of two vortices rotating clockwise separated by a secondary envelope. In Figure 8F (*t*’ = 100), the tail, constituting one roll, is separated completely from the head of V2/+ and the cluster disappears.

The concept of thin film can be extended to the cluster envelope. This film separates the clockwise vortices from the membrane and the surrounding electrolyte. Along the membrane surface, it spreads over a width smaller than the non-equilibrium EDL one. The presence of this film means that a kind of upstream electro-osmotic velocity is imposed between the vortex group and the membrane. However, the immobility of the cluster is not related to this velocity but to the stability of the non-equilibrium EDL.

In the second series (Figure 9), V1/− is initially located between the two rolls V1/+ (*z* = 2.1) and V2/+ (*z* = 2.35). Contrary to Figure 8A, a continuous path of negative ${\left(\vec{F}{}^{Tot}\right)}_{{}_{z}}$ extends along the membrane over the vortex group. Despite this path, no SSL envelops the vortex group because the tops of V1/+ and V2/+ are too separated, allowing V1/− to spread far from the membrane interface. V1/+ and V2/+ get closer (Figure 9B) and the SSL between the vortices are more and more tilted owing to the profile of ${\left(\vec{F}{}^{Tot}\right)}_{{}_{z}}$ (see comments in Figure 7 and Figure 8).

At the top of V1/+ and V2/+, the imposed main flow drives the fluid located close to the vortices in the downstream direction by means of the viscous force. At the same time, in the inter-vortex region, the longitudinal pressure force pushes the fluid upstream. Therefore, at the outer edge of the inter-vortex region, the SSL changes direction and the two points of the same SSL, on both sides of the neck, are close to each other. This behavior is all the more pronounced given that the two vortices are close to each other and the magnitude of the negative pressure spot ${\left(\vec{F}{}^{Tot}\right)}_{{}_{z}}$ increases. Therefore, the SSL join at the upper region of the roll group at *t*’ = 40 (Figure 9C). This induces a displacement of V1/− to the membrane and the presence of an envelope around the vortex group which becomes a cluster. At the same time, the roll (V0/+ located at *z* = 1.6) upstream of the cluster moves closer with a decreasing volume. From *t*’ = 40 to 80 (Figure 9C–E), the tail of V2/+ extends as the downstream vortex goes away and a charge hump appears at *x* = 2.5 as explained in Figure 8C. Later, the cluster breaks, V0/+ fuses with V1/+, V2/+ and its tail becomes independent, and the negative layer ${\left(\vec{F}{}^{Tot}\right)}_{{}_{z}}$ in the inter-vortex region is transformed into a spot. It seems that a cluster including three clockwise vortices is not stable or may only exist during a very short time.

### 3.3. Dynamic Streamlines of Fluid Flow and Ionic Transfer

A fully Lagrangian approach to the fluid flow and ion transfer is necessary to investigate the mixing mechanisms in unsteady flow. In this section, the dynamic streamlines (DSL) display the real trajectories of the fluid and ions, and point out that the vortices visualized by the SSL are fictitious. In this way, for instance, the DSLs show that, in a periodic regime, the vortices and the region far from the membrane, i.e., the diffusion layer containing high ion concentration, are linked by a mechanism of fluid flux through the vortices. However, in an aperiodic regime, the unsteady size of the inter-vortex regions and the vortices promotes fluid exchange with the diffusion layer and enhances the efficiency of the mixing. The same parameter values as in Section 3.2 are used.

#### 3.3.1. Fluid DSL in Periodic Regime ($\Delta \Phi_{}=22$)

**Figure 10:**
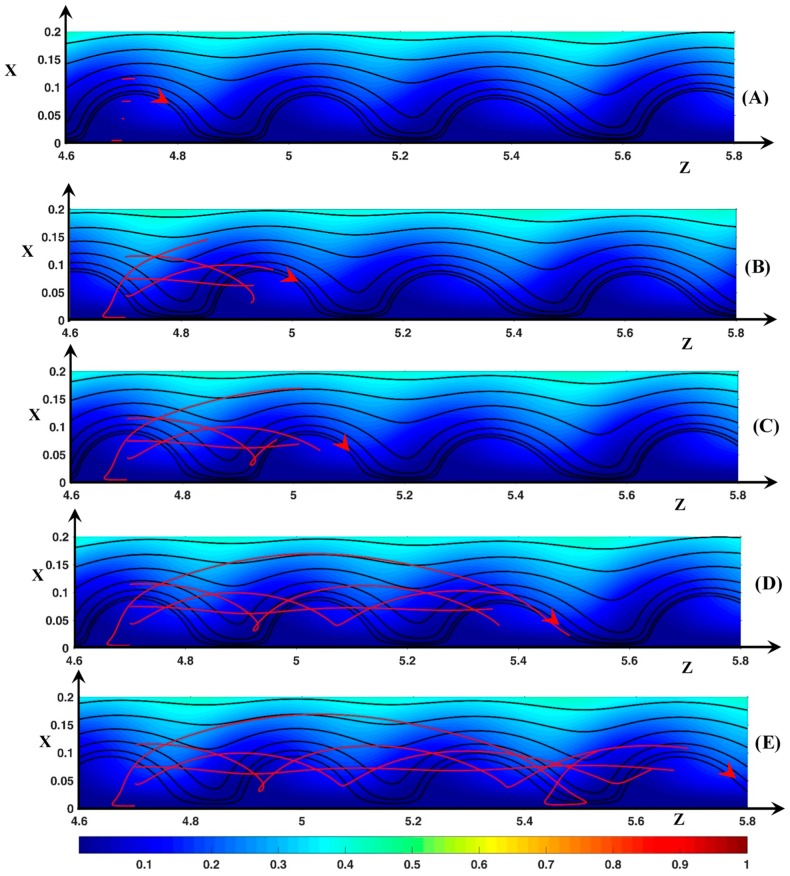
Fluid trajectories. Black lines: static streamlines starting from the inlet. Red lines: dynamic streamlines starting from *z*_o_ = 4.7 (*x*_o_ = 0.005, 0.045, 0.075, 0.115). Color plot: cation concentration. The red arrow represents the rotation direction of the vortices and the position of the vortex at time *t*’ (the trajectories start at *t*’ = 0 inside this vortex). (**A**) *t*’ = 5; (**B**) *t*’ = 70; (**C**) *t*’ =95; (**D**) *t*’ = 195; (**E**) *t*’ = 275. ΔΦ = 22, $\nu =1/3\times 10^{-3}$, $D^{\pm }=1$.

Figure 10 shows four dynamic streamlines at five different times. The line source is located inside a vortex at $z_{o}=4.7$. The vortices move with a longitudinal velocity ($U_{z}^{roll}$) of 36. As in Section 3.2, the time value will be expressed in terms of time step and one assumes that initially *t*’ = 0.

Let us consider the starting point $x_{o}=0.005$. This point is located between the membrane surface and the $E_{c}$ maximum inside the $E_{c}$ spot. At this point, the transverse equilibrium between the electric force and the pressure force is fulfilled. Initially, $U_{z}=-43$ and $U_{x}=-1$. At *t*’ = 10, the fluid begins to move upward as it comes closer to the vortex front. Then $U_{x}$ and $U_{z}$ increases and decreases, respectively. Consequently, at *t*’ = 20 (*x* = 0.033), $U_{x}$ reaches a maximum value of 49 and $U_{z}=24<U_{z}^{roll}$. Then $U_{z}$ increases and becomes greater than $U_{z}^{roll}$. $U_{z}$ decreases first because the fluid element moves away from the $E_{c}$ spot (see Figure 6) and then increases owing to the velocity inlet boundary condition. At *t*’ = 95 (Figure 10C), the fluid is just above the vortex (*x* = 0.17, *z* = 5) with $U_{x}\approx 0$ and $U_{z}=74.5$. During the upward motion, the DSL deviates from the vortex front for two reasons: first $U_{z}<U_{z}^{roll}$ for a period of time and then the angle θ between the membrane surface and the front is lower than $90^{o}$. With regards to the 2nd reason, if the fluid element moves during a time interval Δt upward over the distance $\Delta x=U_{x}\Delta t$, the distance to the front becomes $\Delta z_{2}=U_{x}\Delta t\times \tan\left(\pi /2-\theta \right)$. Finally, the distance from the fluid element to the front is $\Delta z=\Delta z_{1}+\Delta z_{2}$ with $\Delta z_{1}=\left(U_{z}^{roll}-U_{z}\right)\Delta t$ and ∆*z* cancels if $U_{z}=U_{z}^{roll}+U_{x}\times \tan\left(\pi /2-\theta \right)$. If $U_{x}>0$, $U_{z}$ must be much greater than $U_{z}^{roll}$ if θ is small. As *Q* is positive, ${\left(\vec{F}{}^{El}\right)}_{{}_{x}}$ is always negative. In the front region ${\left(\vec{F}{}^{Pr}\right)}_{{}_{x}}>0$ (see Figure 5B). During the fluid rising, $\left|{\left(\vec{F}{}^{Pr}\right)}_{{}_{x}}\right|$ and $\left|{\left(\vec{F}{}^{El}\right)}_{{}_{x}}\right|$ decrease but the pressure contribution is higher than the electric one. Above the vortex, the force balance occurs. However, the force magnitude is very small because *Q* is very small. On the other side of the vortex, ${\left(\vec{F}{}^{Pr}\right)}_{{}_{x}}$ is negative. Therefore, the downward motion of the fluid is accelerated due to the two negative force contributions. The fluid enters the vortex at *t’* = 190 far from the center of rotation and close to the membrane (x=0.03, z=5.47—Figure 10D) (the fluid entrance can be explained by the above relation $\Delta z=\Delta z_{1}+\Delta z_{2}$ with $U_{x}<0$ and θ>π/2). The fluid element continues to move to the membrane surface until the transverse force equilibrium is fulfilled ($U_{x}=0$). Owing to the vortex displacement in the downstream direction, the $E_{c}$ spot (with $U_{z}<0$) moves towards the fluid element. This induces the displacement of the fluid to the front with $U_{x}\approx 0$, so that at *t*’ = 230/240, the fluid element leaves the vortex and then moves upward.

Figure 10 shows that the fluid motion looks like a trochoid around the same vortex. The time and the spatial period are approximately 210 and 0.8, respectively. As $x_{o}$ increases to 0.035, the fluid element is less and less accelerated in the ejection region at the vortex front and spends less time in it. Therefore, the maximal position in the transverse direction decreases, the loop of the trochoid appears more and more early and its size decreases. So that the period decreases to 70 and 0.39 in time and space, respectively.

If the starting point is located at ($x_{o}=0.045\;,\;z_{o}=4.7$), the streamline shape shows another kind of trochoid because the starting point is located above the center of rotation. Initially $U_{x}\approx -5$ and $U_{z}=-9$. The fluid moves upward then downward as explained above. This time, the DSL enters the vortex at *t*’ = 70 at the position (x=0.1, z=4.97) close to the top of the vortex (Figure 10B). During the downward displacement, $\left|U_{x}\right|$ and $U_{z}$ decrease because the fluid element moves closer to the center of rotation. $U_{z}$ becomes lower than $U_{z}^{roll}$ and at *t*’ = 115 the center of rotation catches up with the fluid element. Then the fluid is pushed downstream with an increasing value of $U_{z}$. So, the fluid element stays above the center of rotation. As the vortex front moves closer to the position of the fluid element, the latter leaves the vortex at *t*’ = 160 (x=0.071, z=5.13). The time and spatial periodicity are around 114 and 0.37, respectively.

As *x_o_* increases to 0.09, the transverse amplitude of the trochoid decreases and the periodicity remains equal to about 110 and 0.37 in time and space, respectively. This time periodicity corresponds to the time needed for the vortices to move over the distance between two successive vortex fronts. Outside the vortex ($x_{o}>0.09$), the trajectory is in fact a prolongation of another trajectory beginning upstream $\left({z^{\prime }}_{o},{x^{\prime }}_{o}\right)$ with ${x^{\prime }}_{o}$ lower than the rotation center and at an earlier time given the periodic shape of the trajectory. As a last observation, Figure 10 shows that if $x_{o}=0.075$, the trajectory is linear ($U_{x}\approx 0$). The fluid element remains close to the rotation center and moves with the same velocity as the roll.

Therefore, the figure shows that the real width of the “hydrodynamic instability” layer is much larger than the vortex one. Its outer edge is in contact with the diffusion layer. In the reference frame of the vortices, the trajectories form concentric circles. The rotation center, placed at x≈0.75, is above the SSL vortex one. The fluid exchange between the vortex layer and the diffusion layer is controlled by an inward flux at the rear and an outward one at the front. The trajectories passing close to the membrane are the most efficient for the fluid exchange. This explains the presence of the mixing layer whose width is higher than the SSL vortex one.

**Figure 11:**
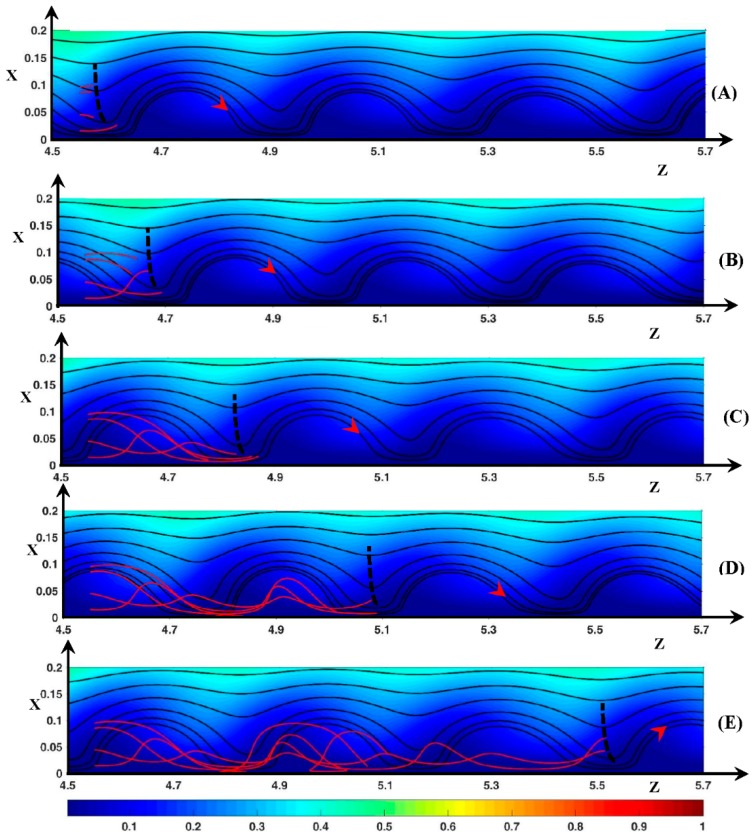
Fluid trajectories. Black lines: static streamlines starting from the inlet. Red lines: dynamic streamlines starting from *z*_o_ = 4.55 (*x*_o_ = 0.015, 0.045, 0.085, 0.095). Black dashed line: curve defined by *U_x_* = 0. Color plot: cation concentration. The red arrow represents the rotation direction of the vortices and the position of the vortex at time t’ (this vortex is downstream the trajectories source at *t*’ = 0). (**A**) *t*’ = 10; (**B**) *t*’ = 35; (**C**) *t*’ = 80; (**D**) *t*’ = 155; (**E**) *t*’ = 275. ΔΦ = 22, $\nu =1/3\times 10^{-3}$, $D^{\pm }=1$.

Now if $z_{o}=4.55$, i.e., upstream of the vortex front, the DSL shape is not always a trochoid because the fluid element can remain inside the inter-vortex region as shown in Figure 11. As an example, let us consider the starting position ($x_{o}=0.015$, $z_{o}=4.55$). The point is located inside the $E_{c}$ spot. Initially, $U_{x}=-6$ and $U_{z}=87>U_{z}^{roll}=U_{z}^{spot}$. Therefore, as the DSL gets closer to the front vortex, the fluid element begins to move upward, and $U_{z}$ decreases to a value smaller than $U_{z}^{roll}$ (see the discussion about Figure 10). Consequently, at *t*’ = 35 (Figure 11B), the vertical line defined by $U_{x}=0$ (black dashed line) and the DSL meet at z=4.67 (the fluid element is at x=0.063). This line moves with the same velocity as the vortices. Therefore, the fluid element passes on the other side of this line and moves towards the membrane with an increasing velocity. Then it enters the $E_{c}$ spot, so that $U_{z}$ reaches a value of 85 at *t*’ = 75 (see Figure 11C). Therefore, the fluid element remains in the inter-vortex region (at *t*’ = 275 z=5.5, x=0.06—Figure 11E). This behavior is observed until *x_o_* = 0.065. If $0.075\leq x_{o}\leq 0.095$, i.e., at the outer edge of the inter-vortex region, the trochoid shape is observed but the DSL does not wind around the same roll. Most of the time, the fluid element moves with $U_{z}<<U_{z}^{roll}$ and with $\left|U_{x}\right|$ small enough for the fluid element to enter the rear of the upstream vortex close to the membrane. This is shown for example with $x_{o}=0.095$ in Figure 11C (*x* = 0.014, *z* = 4.76). The loop is located at *z* = 4.8 (Figure 11D). Figure 11E shows that the same fluid element enters the even upstream vortex a second time at *t*’ = 255 (*x* = 0.024, *z* = 5.06). The loop at *z* = 5 belongs to the trajectory beginning at $x_{o}=0.085$. Therefore, the inter-vortex is not connected to the diffusion layer and it has a small role in the mixing process.

#### 3.3.2. Fluid DSL in Aperiodic Regime (∆Φ = 27)

**Figure 12:**
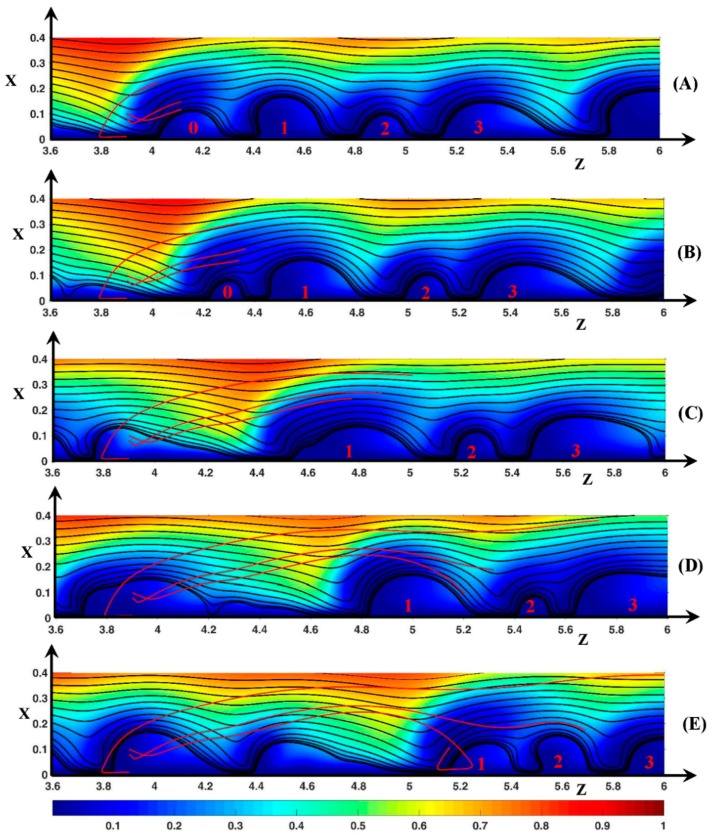
Fluid trajectories. Black lines: static streamlines starting from the inlet. Red lines: dynamic streamlines starting from *z*_o_ = 3.9 (*x*_o_ = 0.01, 0.08, 0.09). Color plot: cation concentration. Red numbers: vortex numbering. Vortex n°0 is the vortex in which the trajectories source is located at *t*’ = 0. (**A**) *t*’ = 55; (**B**) *t*’ = 85; (**C**) *t*’ = 135; (**D**) *t*’ = 190; (**E**) *t*’ = 245. ΔΦ = 27, $\nu =1/3\times 10^{-3}$, $D^{\pm }=1$.

In Figure 12 the source is located inside the vortex V0. Initially, the front of V0 and of the downstream vortex V1 are at *z* = 3.7 and *z* = 4.25, respectively. Until *t*’ = 25, the volume of V0 is unchanged and its height is around 0.18. Then its volume decreases during the merging process with V1. The front of V0 moves with a longitudinal velocity of 66.7 until *t*’ = 85. Furthermore, $U_{z}^{roll}$ is smaller than the fluid inlet velocity at *x* = 0.18 (*U_z_* = 74). The front of V1 remains at the same place in the time interval. Two starting points, (*x_o_* = 0.01, *z_o_* = 3.9) and (*x_o_* = 0.1, *z_o_* = 3.9), are chosen for the discussion because they give rise to two kinds of motion.

In the first case, the fluid element moves first upstream along the membrane to the vortex front, then moves upward in the front region at *t*’ = 25 (*x* = 0.01, *z* = 3.8) and finally passes V0 at *t*’ = 85, as explained in Section 3.3.1. The height difference between V0 and V1 makes the SSL tilted to the upward direction for a long time, such that at *t*’ = 85 the fluid element is at a transverse position much greater than the height of V0. Reaching V1, the fluid leaves the instability region (Figure 12C). The electric force acts no more. The fluid moves mainly in the longitudinal direction with $U_{z}\approx 120>>U_{z}^{roll}$. Therefore, the addition of the high jump at *t*’ = 25 and of the increase of the height of V1 leads to the ejection of the fluid outside the vortex layer.

On the contrary, the fluid remains close to the instability layer if *x*_o_ = 0.1. The source is located above the center of rotation of V0. Therefore, the fluid element leaves the vortex outside the fluid ejection region as explained in Section 3.3.1 (see Figure 12A). The fluid element is just above V0 (Figure 12B) and then above V1 at *t*’ = 135 because $U_{z}>U_{z}^{roll}$ (Figure 12C). It reaches its maximum transverse position *x* = 0.25 at *z* = 4.77. From *t*’ = 135 to 190, as the fluid element overtakes V1, the height of V1 increases to 0.18 and its shape becomes symmetric. The static streamlines are more and more directed downward near the rear region of V1 (see Section 3.2.1 ∆Φ = 23) and the fluid element is driven towards the membrane. For the same reason as explained in the case of ∆Φ = 22, the fluid element must enter the vortex V1 and the loop of the trochoid begins at *t*’ = 210 (*x* = 0.03, *z* = 5.2).

As *x*_o_ decreases from 0.1, the DSL reaches a higher transverse position during the fluid excursion, i.e., the fluid explores the diffusion layer more deeply, and it enters the vortex layer at a higher distance from the source position. This means that the high transverse amplitude of the trajectory and the large dispersion of the DSL allow a fluid exchange between different distant vortices in contrast to the periodic regime.

**Figure 13:**
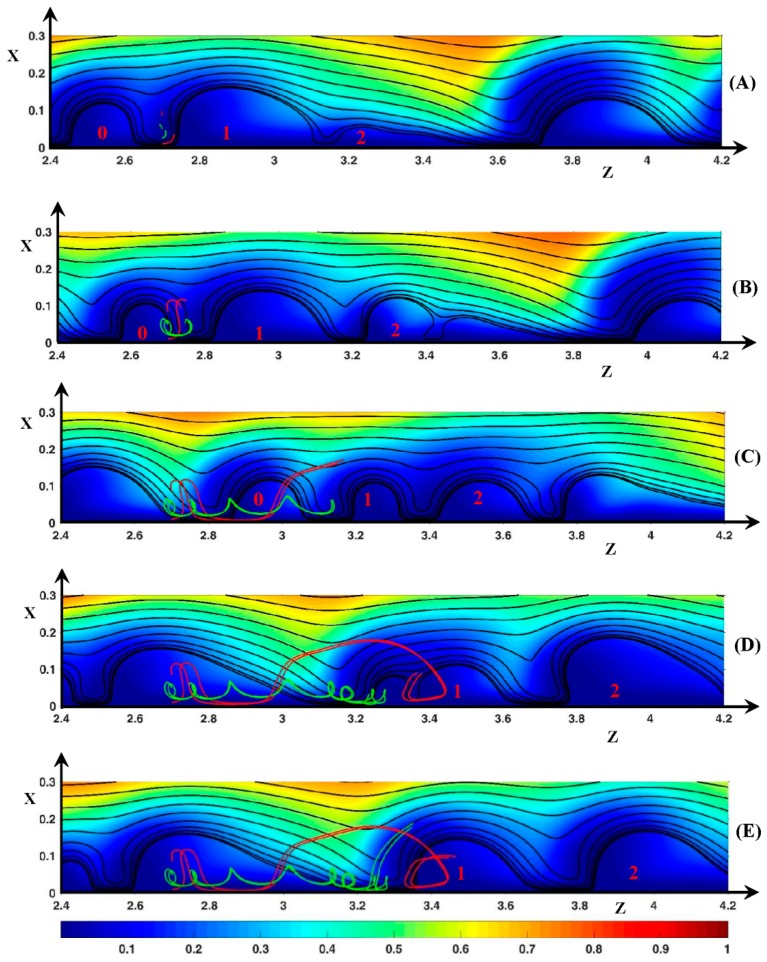
Fluid trajectories. Black lines: static streamlines starting from the inlet. Red and green lines: dynamic streamlines starting from *z*_o_ = 2.7 (*x*_o_ = 0.01, 0.025, 0.05, 0.085). Color plot: cation concentration. Red numbers: vortex numbering. Vortex n°0 is the vortex upstream the trajectories source location at *t*’ = 0. Vortex n°1 is the vortex downstream the source. (**A**) *t*’ = 5; (**B**) *t*’ = 45; (**C**) *t*’ = 135; (**D**) *t*’ = 220; (**E**) *t*’ = 245. ΔΦ = 27, $\nu =1/3\times 10^{-3}$, $D^{\pm }=1$.

Figure 13 displays the fluid trajectories when the source is located at *z*_o_ = 2.7 between V0/+ and V1/+ (see Figure 13A). Between these rolls, a counter-clockwise roll (V/−) is located at x≈0.04. During the rolls’ displacement, the inter-vortex region between V0/+ and V1/+ expands and V/− disappears at *t*’ = 55. At *t*’ = 135 (Figure 13C), V1/+ stops to move downstream ($z_{front}=3.2$), V0/+ moves closer to V1/+ and the roll V/− appears one more time between these two vortices, which merge at *t*’ = 225 (Figure 13D).

Let us consider the first trajectory *x*_o_ = 0.01 (red trajectory). This starting point is located inside the *E*_c_ spot close to the membrane, i.e., in the fluid film below V/−. If *t*’ < 80 (*z* < 3), the fluid trajectory is similar to the trajectory beginning at *x_o_* = 0.015, shown in Figure 11. The fluid element remains in the thin film surrounding V/− in the inter-vortex region. However, the fluid element passes above V/− during the first upward period and leaves for a very short time the fluid film at the outer edge of the inter-vortex region. This means that V/− is also isolated from the neck, a behavior which the SSL cannot not reveal (see Figure 8 and Figure 9). At *t*’ = 100 (*x* = 0.12, *z* = 3), the fluid element reaches the region where *U_x_* is small. However, the fluid element does not cross the boundary *U_x_* = 0 because the inter-vortex region expands. Therefore, the fluid element continues to move upward and leaves the inter-vortex region with an increasing longitudinal velocity, which becomes higher than $U_{z}^{roll}$ (see Figure 13C). As the size of V1/+ increases when *t*’ > 135, the DSL enters the V1/+ rear at *t*’ = 175 as explained previously and then the fluid trajectory makes a loop inside V1/+.

For the source point *x*_o_ = 0.025 (green trajectory), the fluid element is initially inside V/−. The vortex is isolated from the neighborhood. So, until *t*’ = 55, the fluid element remains inside V/− and rotates counter-clockwise two times. At *t*’ = 60, V/− disappears; therefore, until *t*’ = 125 (see Figure 13C), the fluid trajectory oscillates in the transverse direction as explained in Section 3.3.1. At *t*’ = 135, V/− appears, and the fluid element makes two counter-clockwise rotations until *t*’ = 180 (*x* = 0.012, *z* = 3.2). The inter-vortex region between V0/+ and V1/+ is so small that the rear of V0/+ passes the fluid element during its upward motion at *t*’ = 200 (*x* = 0.045, *z* = 3.25). The fluid consequently moves upstream inside V0/+ to the front (Figure 13D), then it moves upward during the merging of V0/+ with V1/+. The volume of the new vortex increases. Therefore, as the fluid element moves with $U_{z}>U_{z}^{roll}$ at *t*’ = 245, it must enter the new vortex by the rear later on and must then begin a loop motion.

The two last Figures show that several kinds of trajectories follow one another owing to the vortex dynamics. Therefore, the trajectories playing a small role in the exchange between the vortex layer and the diffusion layer have a finite lifetime. The inter-vortex regions exchange fluid with the vortices. As in the periodic mode, the passage through the vortices plays a key role in this exchange and enhances the ion exchange. The vortices trap the fluid elements by means of their changing shape. The counter-clockwise vortices do not exchange fluid through its boundary.

#### 3.3.3. Ionic DSL in the Periodic Regime ($\Delta \Phi_{}=22$)

In this section, the ion transfer in the aperiodic regime is not investigated because the anions are simply ejected from the vortex region if ∆Φ > 24, and the cations follow the same kind of trajectories as in periodic regime.

**Figure 14:**
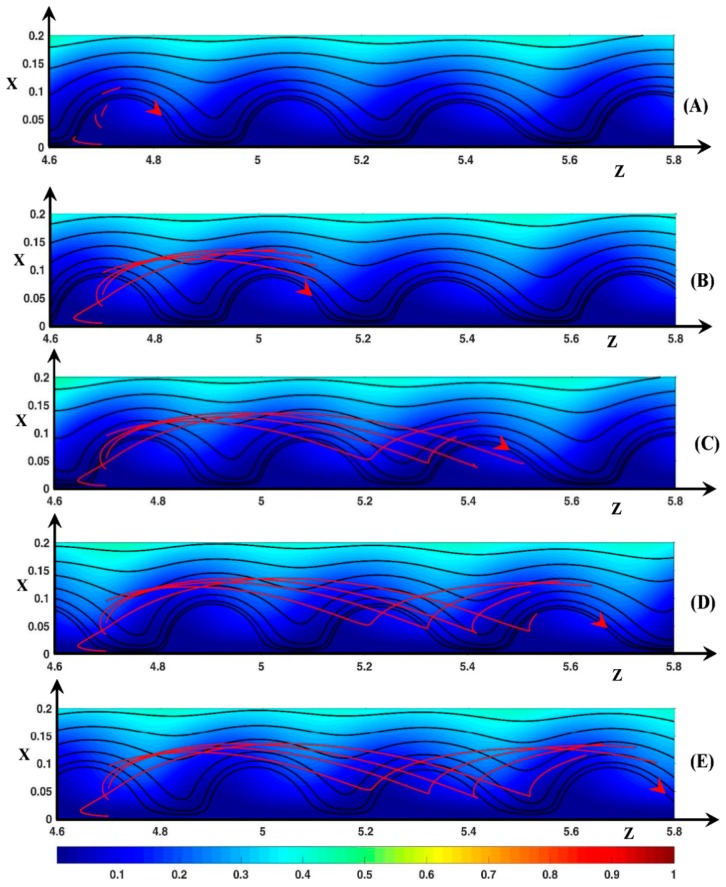
Fluid and anionic trajectories. Black lines: fluid static streamlines starting from the inlet. Red lines: anionic dynamic streamlines starting from *z*_o_ = 4.7 (*x*_o_ = 0.005, 0.035, 0.055, 0.085). Color plot: cation concentration. The red arrow represents the rotation direction of the vortices and the position of the vortex at time *t*’ (the trajectories start at *t*’ = 0 inside this vortex). (**A**) *t*’ = 10; (**B**) *t*’ = 95; (**C**) *t*’ = 210; (**D**) *t*’ = 250; (**E**) *t*’ = 275. ΔΦ = 22, $\nu =1/3\times 10^{-3}$, $D^{\pm }=1$.

Let us consider the anionic motion when the starting longitudinal position is $z_{o}=4.7$, i.e., inside a vortex as shown in Figure 14. It is recalled that the ion velocity ($\vec{U}{}^{\pm }$) is computed by means of the ion flux density ($\vec{J}{}^{\pm }$) (see Section 2.2.2). 

Consider first the case $x_{o}=0.005$. At this distance from the membrane, the negative Fickian contribution ${\left(\vec{J}{}_{Fi}^{-}\right)}_{{}_{x}}$ balances the positive electric one ${\left(\vec{J}{}_{Em}^{-}\right)}_{{}_{x}}$. The convection ${\left(\vec{J}{}_{Co}^{-}\right)}_{{}_{z}}$ and the concentration gradient ${\left(\vec{J}{}_{Fi}^{-}\right)}_{{}_{z}}$ push the anions to the vortex front. At the beginning of the rising period (Figure 14A), the anions are pushed transversely by convection because ${\left(\vec{J}{}_{Fi}^{-}\right)}_{{}_{x}}=-{\left(\vec{J}{}_{Em}^{-}\right)}_{{}_{x}}$. Then around the position (*x* = 0.1, *z* = 4.8), the electro-migration contribution becomes dominant with ${\left(\vec{J}{}_{Fi}^{-}\right)}_{{}_{x}}\approx -{\left(\vec{J}{}_{Co}^{-}\right)}_{{}_{x}}$. Finally at *t’* = 95 Figure 14B, the ions reach the maximal transverse position with the balance ${\left(\vec{J}{}_{Fi}^{-}\right)}_{{}_{x}}\approx -{\left(\vec{J}{}_{Em}^{-}\right)}_{{}_{x}}\cdot {\left(\vec{J}{}_{Co}^{-}\right)}_{{}_{x}}\approx 0$) and with $U_{z}^{-}>U_{z}^{roll}$. In the longitudinal direction, the ions move by convection. During the downward motion (see Figure 14C), the ions enter the same vortex at $t{}^{\prime }\approx 210$ and get closer to the vortex rotation center. The ions are pushed to the membrane by convection and diffusion and with $U_{z}^{-}<U_{z}^{roll}$. At $t{}^{\prime }\approx 210$, the anions arrive at the same transverse position as the rotation center. $\left(\vec{J}{}_{Co}^{-}\right)$ becomes negligible and $\left(\vec{J}{}_{Fi}^{-})\approx -(\vec{J}{}_{Em}^{-}\right)$, i.e., the anions arrive at a stagnation point. Therefore, after a while the anions cross over to the other side of the rotation center, then they begin an upward motion and leave the vortex a second time. As *x_o_* increases from 0.035 to 0.085, the ions deviate from the ejection region as they leave the vortex. Consequently, the transverse amplitude of their trajectory decreases, and the cusps move upstream.

**Figure 15:**
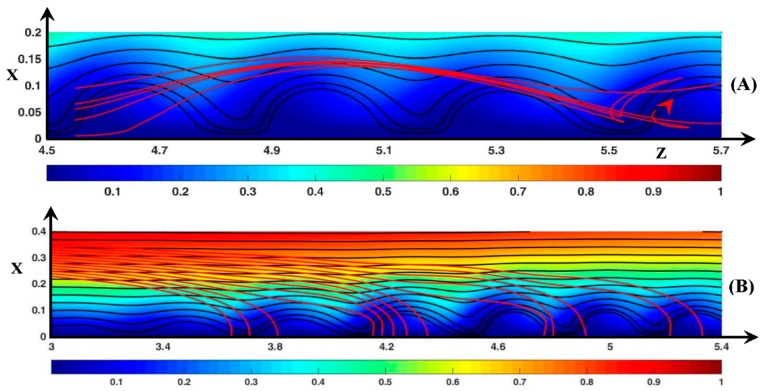
Fluid and ionic trajectories. Red lines: (**A**) anionic dynamic streamlines starting from *z*_o_ = 4.55 (*x*_o_ = 0.005, 0.045, 0.055, 0.065, 0.1). The red arrow represents the position of the vortex at time *t*’ (this vortex is downstream the trajectories source at *t*’ = 0); (**B**): cationic dynamic streamlines starting from *z*_o_ = 3, *x*_o_ = [0.23, 0.35]. Black lines: fluid static streamlines starting from the inlet. Color plot: cation concentration. *t*’ = 275. ΔΦ = 22, $\nu =1/3\times 10^{-3}$

Figure 15A displays the anionic DSL when the anion source is located between two vortices (*z*_o_ = 4.55). Two families of trajectory can be observed. The first one is identical to the trajectories shown in Figure 14 if *x_o_* lies between 0.035 and 0.055. However, the stagnation point is replaced by an elongated and tilted loop because the anions reach a transverse position lower than the rotation center one and move to the vortex front by diffusion ${\left(\vec{J}{}_{Fi}^{-}\right)}_{{}_{z}}$ and convection ${\left(\vec{J}{}_{Co}^{-}\right)}_{{}_{z}}$. At higher values of *x*_o_, $U_{z}^{-}$ is always positive because the DSL never enter the vortex owing to higher fluid velocity and to the positive contribution of ${\left(\vec{J}{}_{Fi}^{-}\right)}_{{}_{z}}$. The sign of ${\left(\vec{J}{}_{Fi}^{-}\right)}_{{}_{z}}$ is positive because the anion position is always downstream of the location of the high ion concentration region at the vortex rear. The consequence is that the anions move faster than the vortices along the membrane if $x_{o}\geq 0.075$.

Therefore, the longitudinal Fickian contribution is of great importance to the Lagrangian anionic motion. The two last Figures also show that despite the electric force directed upward, the anions remain in the layer explored by the fluid DSL. At the outer-edge of this layer, the Fickian diffusion balances the electro-migration and keeps the anions leaving the region.

In regards to the cations (Figure 15B), the dynamic streamlines are computed with the vertical source (*z*_o_ = 3, $0.23\leq x_{o}\leq 0.35$). This time, the electro-migration flux is directed downward like the Fickian one. ${\left(\vec{J}{}_{Em}^{+}\right)}_{{}_{x}}$ is the main contribution to the cation motion towards the membrane, and Figure 15B shows that the cations move monotonically. At the initial time, cation motion is mostly longitudinal ($U_{z}^{+}>U_{x}^{+}$). As they get closer to the membrane, the DSL become vertical because ${\left(\vec{J}{}_{Em}^{+}\right)}_{{}_{x}}$ increases and becomes dominant. The convective contribution has the same order of magnitude as the electro-migration one in the region around the outer edge of the vortex layer and it leads to the increase of the cationic flux. The DSL form four groups at the vicinity of the membrane because they reach the membrane through the inter-vortex region and the rear of the rolls, i.e., regions where the transverse cationic flux is important.

## 4. Conclusions and Summary

In this work, the analysis of electro-convective instabilities in a laminar boundary layer along a cation exchange membrane is presented. The periodic and aperiodic modes were studied. In particular, for the latter mode, vortex association/dissociation and fusion, as well as time evolution of cluster and counter-clockwise vortex, were investigated. The influence of the instability structure and dynamics on the fluid and ion transfer was also investigated by means of a Lagrangian approach. The aperiodic mode is sensitive to the value of the parameters $D^{\pm }$ and *ν*. Despite this observation, the present study was performed mainly with $D^{\pm }=1$ and $\nu =1/3\times 10^{-3}$.

The vortex structure and dynamics were analyzed by considering the spatial distribution of the longitudinal component of the electric and pressure force. This distribution is composed of a layer along the membrane inside the non-equilibrium EDL and of two spots on both sides of the vortex fronts and above the force layer. These two structures undergo a change of sign at the front and the rear of the vortices. The link between a spot and a layer of negative sign plays a key role in the vortex dynamics and in the birth of counter-clockwise vortices and clusters. This link bends the streamlines in the upward direction and induces a neck at the outer edge of the inter-vortex region where the fluid is pushed downward by the forced flow. The counter-clockwise vortices occur in the inter-vortex region over which the negative spot is located. This vortex is isolated from the neighboring clockwise vortices and the membrane by a thin fluid film coming from the neck. The clockwise vortices associate into a cluster when the neck closes, and a continuous path of negative longitudinal force occurs over the vortex group along the membrane. In this case, a thin film also wraps the vortex group, which is therefore separated from the membrane and the boundary layer. The counter-clockwise vortices are excluded from the clusters in contact with the membrane during the clustering process. The upstream fluid motion in the film along the membrane plays a similar role as the electro-osmotic boundary conditions.

The real motion of the fluid, as well as the ions, must be computed by considering the unsteadiness of the velocity and flux fields. In the periodic mode, the fluid trajectories passing through a vortex, computed with the SSL, form a trochoid. The SSL vortices are fictitious. In the vortex reference frame, these DSLs form rolls whose rotation center is above the SSL vortex center and whose size is much greater. The real vortex layer is therefore in contact with the diffusion layer containing high ion concentration and it controls the mixing layer expansion. This is not the case for the inter-vortex region. Surprisingly, the anions follow the same kind of trajectory with roughly the same transverse amplitude. The Fickian diffusion plays a key role in the loop formation and in the transverse amplitude. The outer edge of the region explored by the anions corresponds to the balance between the electro-migration and the Fickian diffusion fluxes in the transverse direction. Further away from the instability threshold, the electric field is high enough for the anions to be ejected from the vortex layer.

For the aperiodic mode, the real instability layer visualized by the DSL is also much larger than the SSL rolls. Its size increases with ∆Φ, imposing the upward displacement of the diffusion layer. In this case, fluid exchange is performed between distant SSL vortices. The transverse amplitude of the trajectories is high enough for the mean Lagrangian velocity to be much greater than the SSL roll one. The size increase allows the rolls to attract and trap the fluid elements. It is also observed that the inter-vortex regions contribute to the mixing process. The variation of the inter-vortex length destabilizes the fluid trajectory leading to the fluid ejection outside the SSL vortex layer and initiating a trochoid-like trajectory.

## Figures and Tables

**Table 1:** Width cation boundary layer defined by c^+^ = 0.6 at the outlet of the domain. 1st row: published values (Nikonenko et al. [28] and Urtenov et al. [41]). 2nd row: this work.

$x_{c^{+}=0.6}$	100 µm [41]	140 µm [41]	90 µm [28]
$x_{c^{+}=0.6}$	100 µm	150 µm	75 µm

**Table 2:** Parameter values of the correlation $\delta_{v}/L_{m}=a{\left(\Delta \Phi_{v}^{2}/\langle U_{z}\rangle \right)}^{1/3}+b$. 1st and 2nd columns: published values. 3rd columns: this work.

	Kwak et al. [18]	Urtenov et al. [41]	This Work
a	0.0101	0.0196	0.0194
b	−0.1714	−0.1084	−0.0107

**Figure A1:**
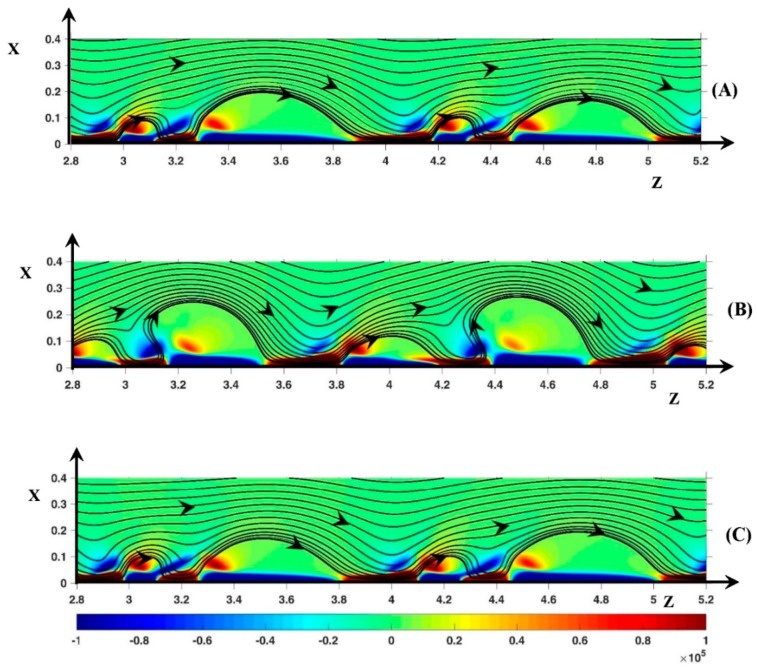
Temporal evolution of the vortices. (**A**) *t*’ = 0; (**B**) *t*’ = 60; (**C**) *t*’ = 90. Color plot: ${\left(\vec{F}{}^{Tot}\right)}_{z}$. Black lines: static streamlines starting from the inlet. ΔΦ = 35, $\nu =10^{-3}$, $D^{\pm }=1$.

## Abbreviations

PNP	Poisson–Nernst–Planck
EDL	Electric double layer
ESC	Extended space charge
SSL	Static streamline
DSL	Dynamic streamline
**Symbols**	
A	Surface of the domain
$c^{\pm }$, $c_{o}^{}$, $c_{\mathrm{int}erf}^{+}$	Cation and anion concentration, bulk salt concentration, cation concentration at the membrane surface
*e*	Elementary charge
$E_{c}$	Kinetic energy
$D^{\pm }$, $D_{ref}$	Cation and anion diffusion coefficient, reference diffusion coefficient
$\left(\vec{F}{}^{El}\right)$, $\left(\vec{F}{}^{Pr}\right)$, $\left(\vec{F}{}^{Tot}\right)$	Electric force, Pressure force, total force
$k_{B}$	Boltzmann constant
*I*, *I_m_*, *I_lim_*	Mean current density, current density at the membrane surface, limiting current
${\vec{J}}^{\pm }$, ${\vec{J}}_{Co}^{\pm }$, ${\vec{J}}_{Em}^{\pm }$, ${\vec{J}}_{Fi}^{\pm }$	Cation and anion flux, convective, electro-migration and Fickian flux contribution
$L_{x}$, $L_{z}$, $L_{m}$	Longitudinal and transverse domain length, inter-membrane distance
*P*, *P_o_*	Pressure, osmotic pressure
*Pe*	Peclet number
*Q*, $Q_{\max}$	Charge density, maximum charge density in the ESC
*t*, *t_diff_*, δt	Time, diffusional time, time step
*T*	Temperature
$\vec{U}$, ${\vec{U}}^{\pm }$, *U_slip_*, $U_{z}^{roll}$, $U_{z}^{spot}$	Fluid velocity, cation and anion diffusional velocity, imposed velocity at the upper edge of the domain, longitudinal vortex velocity, longitudinal velocity of the kinetic energy spot
$z^{\pm }$	Cation and anion charge
**Greek Symbols**	
$\delta_{v}$	Vortex height
$\varepsilon_{o}$,$\varepsilon_{r}$	Vacuum and relative permittivity
$\lambda_{d}$	Debye length
μ	Dynamic viscosity
ν	Debye length to the longitudinal domain length ratio
$\rho_{f}$	Fluid density
θ	Local angle between the membrane surface and the vortex front
Φ,$\Phi_{v}$,$\Phi_{T}$	Electric potential, potential in the vortex layer, thermodynamic potential
Ω	Domain

## Appendix A. Influence of the Length Ratio on the Instability Modes

If ($D^{\pm }=1$, $\nu =10^{-3}$), the periodic instability, without roll pair formation, occurs in the range 23 ≤ ΔΦ ≤ 25. Aperiodic mode appears at ΔΦ=27. For this potential drop value, rolls of different sizes cross the domain. Vortex association and dissociation are also observed from time to time. However, a new kind of periodicity takes place at ΔΦ≥30. Figure A1 shows the time evolution of the vortex structure at ΔΦ=35.

Three steps have been observed. Every other roll is in the same phase and the rolls between are in another one, that is two phases coexist. The Figures show clearly that, at the vortex front and rear, the SSL orientation is related to the unsteady link between a spot and a layer of longitudinal force of the same sign. Let us consider the smallest rolls at *z* = 3.1 and 4.3 in Figure A1A. As a first step, these rolls, of initial size 0.2 × 0.11 in the *z* and *x* direction, respectively, move to *z* = 3.2 and 4.4 and increase in volume (Figure A1B). At the beginning, the negative spot ${\left(\vec{F}{}^{Tot}\right)}_{{}_{z}}$ is transformed into a tilted layer in the downstream inter-vortex region. As the volume increases (Figure A1B), the SSL turn progressively to the upstream direction at the roll front, and a counter-clockwise vortex in the upstream inter-vortex region appears (*z* = 3.05 and 4.3). It disappears in the next step. This first step lasts 60*δt*. In a second step (Figure A1C), the roll volume decreases and becomes elongated and symmetric (*z* = 3.4 and 4.6). The elongation occurs at the rear, the front remaining roughly at the same place. The roll dimension is about 0.6 × 0.16. This step lasts 30*δt*. In the last step, the roll volume continues to decrease so that the rolls return to their initial form. This third step lasts 80*δt*. As every second roll is in step N, the other rolls are in step N − 1 or N + 1. However, in the same roll family, a small phase shift in the shape evolution may occur from one roll to another and the growth amplitude may also differ. But the mechanism remains the same.
